# Effects of polymorphic cytochrome *P450 2A6* genotypes on chemoprevention against colorectal tumors in single Japanese cohort using daily low-dose aspirin: insights into future personalized treatments

**DOI:** 10.1186/s40780-021-00209-8

**Published:** 2021-07-01

**Authors:** Hiroshi Yamazaki, Makiko Shimizu, Takahiro Otani, Ami Mizugaki, Kanae Mure, Sadao Suzuki, Hideki Ishikawa

**Affiliations:** 1 Laboratory of Drug Metabolism and Pharmacokinetics, Showa Pharmaceutical University, 3-3165 Higashi-tamagawa Gakuen, Machida, Tokyo, 194-8543 Japan; 2 Department of Public Health, Nagoya City University Graduate School of Medical Sciences, Nagoya, Aichi Japan; 3 Department of Public Health, Wakayama Medical University School of Medicine, Wakayama, Japan; 4 Department of Molecular-Targeting Cancer Prevention, Kyoto Prefectural University of Medicine, Sakyo-ku, Kyoto, Japan

**Keywords:** CYP2A6, Acetylsalicylic acid, Chemoprevention, Ethnic difference

## Abstract

**Background:**

A chemopreventive effect of low-dose aspirin against colorectal tumors was previously found in participants of two Japanese multicenter, double-blind, randomized, placebo-controlled clinical trials investigating the effects of daily aspirin (100 mg/day) for 0.7–2 years on tumor recurrence in colorectal cancer patients whose tumors were excised endoscopically.

**Methods:**

In the current study, chemopreventive data from single-center subsets having daily aspirin (100 mg/day) were reanalyzed with respect to variations in polymorphic cytochrome P450 2A6 (CYP2A6). From the J-CAPP study, 56 of 311 participants (47 men, 9 women; excluding patients with familial adenomatous polyposis) were genotyped for CYP2A6*1, *4 (whole-gene deletion), *7 (amino acid substitution), and *9 (upstream mutation), and from the J-FAPP IV study, 81 of 102 participants (43 men, 38 women; including patients with familial adenomatous polyposis) were also genotyped.

**Results:**

The chemopreventive effects of daily aspirin were found to be inversely dependent on the predicted enzyme activity of the CYP2A6 phenotype [based on normal genotypes (CYP2A6*1/*1,*7,*9) and impaired genotypes (CYP2A6*4,*7,*9/*4,*7,*9 and CYP2A6*1/*4)] among a nonsmoker Japanese cohort without familial adenomatous polyposis.

**Conclusions:**

The *CYP2A6* wild-type allele could be a candidate biomarker for reduced chemopreventive effects of daily aspirin in a population with wide-ranging CYP2A6 phenotypes with a high frequency of impaired activities resulting from variations and whole-gene deletions. The *CYP2A6* genotypes could be applicable to future personalized treatments for colorectal tumor chemoprevention with daily aspirin.

## Background

Epidemiological studies have shown that cigarette smoking and the consumption of meat or high-fat products are positively associated with colorectal tumor risk [1–4]. Indeed, these findings and human biomonitoring studies indicate that heterocyclic amines and *N*-nitrosamines in meat-derived products may play important roles in colorectal carcinogenesis [5]. Because cytochrome P450 2A6 (CYP2A6) mediates nicotine oxidation and the metabolic activation of tobacco-related procarcinogens [6, 7], the involvement in tumor development of polymorphic CYP2A6 with impaired activities resulting from whole-gene deletion of *CYP2A6* was postulated [8].

**Fig. 1:**
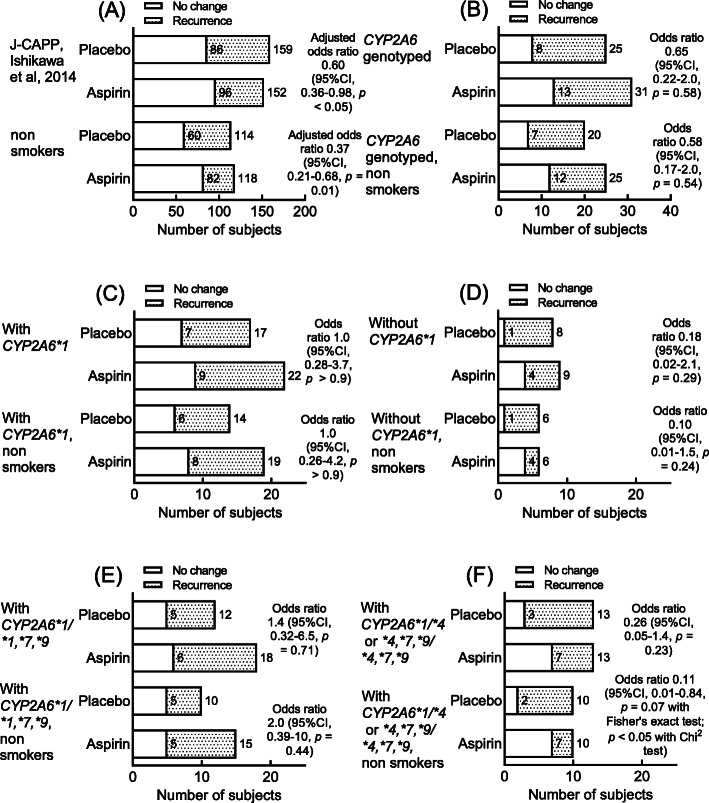
Effects of *CYP2A6* haplotypes and genotypes on aspirin chemoprevention for colorectal tumor recurrence in the total cohort and the nonsmoker subset of Japanese J-CAPP study participants. Data shown in Panel A of adjusted odds ratios by sex, age, and the number of tumors prior to the trial were taken from Ishikawa et al. [9]. The preventive effects of aspirin were evaluated based on the recurrence of polyps observed in 2 years in the J-CAPP study. Odds ratios are shown with respect to the reference (placebo) group

A marked chemopreventive effect of daily low-dose aspirin on the development of colorectal tumors was shown in a Japanese cohort, as shown in Fig. 1a using reported data [9]. Interestingly, subgroup analysis in that report revealed that the use of aspirin in smokers resulted in an increased risk of colorectal tumors, in contrast to the reduced risk in nonsmokers [9]. The abrogation by smoking of colorectal polyp prevention by daily aspirin has been independently postulated in other reports [10–12]. CYP2A6 is known to be a determinant of smoking behavior [8]. The Japanese population has a wide range of CYP2A6 phenotypes resulting in different enzyme activities [13, 14]. However, the relationship between the chemopreventive effects of aspirin and polymorphic CYP2A6 variations with respect to colorectal cancer risk remains unclear.

In the current study, associations between *CYP2A6* genotypes and the chemopreventive effects of aspirin were evaluated based on two independent studies with different endpoints: (1) the recurrence of polyps observed in 2 years and (2) the number of polyps developing to a size of ≥5 mm observed in 8-months. The effectiveness of chemoprevention using daily aspirin to reduce the risk the colorectal tumors was found to be inversely related to the estimated activities of CYP2A6 phenotypes (based on the presence/absence of CYP2A6*1 alleles) among a Japanese cohort without familial adenomatous polyposis. In contrast, when the study group subjects included those with familial adenomatous polyposis, the chemopreventive effects of daily aspirin were present in both those with and without a copy of CYP2A6*1. We report herein that the *CYP2A6* wild-type allele could be a candidate biomarker for reduced chemopreventive effects of daily aspirin in a population with a wide range of CYP2A6 phenotypes including high frequencies of phenotypes with impaired activities caused by variations and whole-gene deletions.

## Methods

The chemopreventive data from single-center subsets having daily aspirin were reanalyzed with respect to variations in polymorphic CYP2A6. The subjects of the current study were 56 of 311 participants (age range 32–70 years, 47 men and 9 women, 19.6% smokers, mainly recruited at the Kyoto Prefectural University of Medicine) of the previously reported multicenter J-CAPP study [9] and 81 of 102 participants (age range 17–61 years, 43 men and 38 women, 8.6% smokers, recruited at Kyoto Prefectural University of Medicine) of the previously completed multicenter J-FAPP IV study [15]. The J-CAPP study was a double-blind, randomized, placebo-controlled clinical trial conducted to investigate the effects of 100 mg/day aspirin for 2 years on tumor recurrence in colorectal tumor patients (excluding individuals with familial adenomatous polyposis) who had had their tumors excised endoscopically. The J-FAPP IV study was also a double-blind, randomized, placebo-controlled trial of colorectal tumor patients, but included cases of familial adenomatous polyposis. J-FAPP IV subjects were also treated with 100 mg/day aspirin in combination with 2 g/day mesalazine for 8 months and had had their tumors excised endoscopically. Signed consent forms and completed questionnaires for this study were collected from all subjects, and data from the two original trials were reanalyzed. This study was approved by the ethics committees of Kyoto Prefectural University of Medicine and Wakayama Medical University.

Genomic DNA was isolated from blood spotted onto storage cards (FTA Elute Sample Collection Cards, GE Healthcare, Tokyo, Japan) using a DNA Extract All Reagents Kit (Thermo Fisher Scientific, Tokyo, Japan). The genotyping of CYP2A6*1, CYP2A6*4 (whole-gene deletion), CYP2A6*7 (amino acid substitution), and CYP2A6*9 (upstream mutation) was performed as described previously [1, 8, 14]. Subjects were assigned to normal or impaired groups based on their *CYP2A6* genotypes [8]: the normal group included those with CYP2A6*1/*1 and those with CYP2A6*1/*7,*9; in contrast, the impaired group consisted of those heterozygous or homozygous for variant alleles CYP2A6*4,*7,*9/*4,*7,*9 and those with CYP2A6*1/*4. The associations between the effects of aspirin and *CYP2A6* genotypes were assessed using odds ratios and 95% confidence intervals with the Fisher’s exact test or χ^2^ tests. All statistical analyses were carried out using the statistical software Prism (GraphPad Software, San Diego, CA, USA) or SAS version 5.0 (SAS Institute, Inc., Cary, NC, USA).

## Results

The chemopreventive effects of daily aspirin on the recurrence of colorectal tumors were analyzed in Japanese cohorts in terms of the polyp recurrence [J-CAPP study, [9]] observed in the 2 years after endoscopic tumor excision. Significant chemopreventive effects of daily aspirin were observed, with odds ratios of 0.60 and 0.37 (95% confidence intervals [CIs], 0.36–0.98 and 0.21–0.68, Fig. 1a) for the total J-CAPP cohort and for the subset of nonsmokers, respectively. A non-significant but favorable odds ratio was also seen in the subset of the J-CAPP cohort who took part in the *CYP2A6* genotyping study (56 subjects, 19.6% smokers, odds ratio 0.65, 95% CI 0.22–2.0, Fig. 1b). In contrast, for those harboring at least one CYP2A6*1 wild-type allele, no chemopreventive effect was found (Fig. 1c). However, the chemopreventive effects of aspirin against colorectal tumor recurrence was suggested to be associated with those who did not carry a wild-type CYP2A6*1 allele (Fig. 1d). Furthermore, chemoprevention using daily low-dose aspirin to reduce the risk of colorectal tumor recurrence tended to be inversely dependent on the predicted enzymatic activities of the CYP2A6 phenotype [based on the genotypes: CYP2A6*1/*1,*7,*9 (normal) and CYP2A6*4,*7,*9/*4,*7,*9 and *1/*4 (impaired)] among a Japanese cohort without familial adenomatous polyposis (Fig. 1e, f). Only minor changes to the odds ratios resulted when they were adjusted by logistic regression for age: age-adjusted odds ratios of 0.25 (95% CI 0.04–1.4) and 0.13 (95% CI 0.02–1.1) were seen versus the unadjusted odds ratios of 0.26 and 0.11 (Fig. 1f) in the total and nonsmoker subsets of the J-CAPP cohort with the impaired *CYP2A6* genotype, respectively. Aspirin chemoprevention for colorectal tumor recurrence was significantly observed (*P* < 0.05) in the male nonsmoker subset of the J-CAPP cohort genotyped for CYP2A6*1/*4 and *4,*7,*9/*4,*7,*9, i.e., the putative impaired phenotype (Table 1)*.*

**Table 1:** Aspirin chemoprevention for colorectal tumor recurrence in a male nonsmoker subset of the Japanese J-CAPP cohort genotyped for CYP2A6*1, *4, *7, and *9

	No change	Recurrence of polyps	Total	Odds ratio (95% CI)	*P* value
CYP2A6*1/*1,*7,*9 (normal genotypes)					
Placebo	2	3	5	2.2 (0.24–24)	*P* = 0.58 with Fisher’s exact test
Aspirin	3	10	13		
CYP2A6*1/*4 and *4,*7,*9/*4,*7,*9 (impaired genotypes)					
Placebo	1	8	9	0.06 (0.005–0.76)	*P* < 0.05 with Fisher’s exact test
Aspirin	6	3	9		

Odds ratios are shown with respect to the reference (placebo) group. *P* for interaction was 0.043 (adjusted for age)

**Fig. 2:**
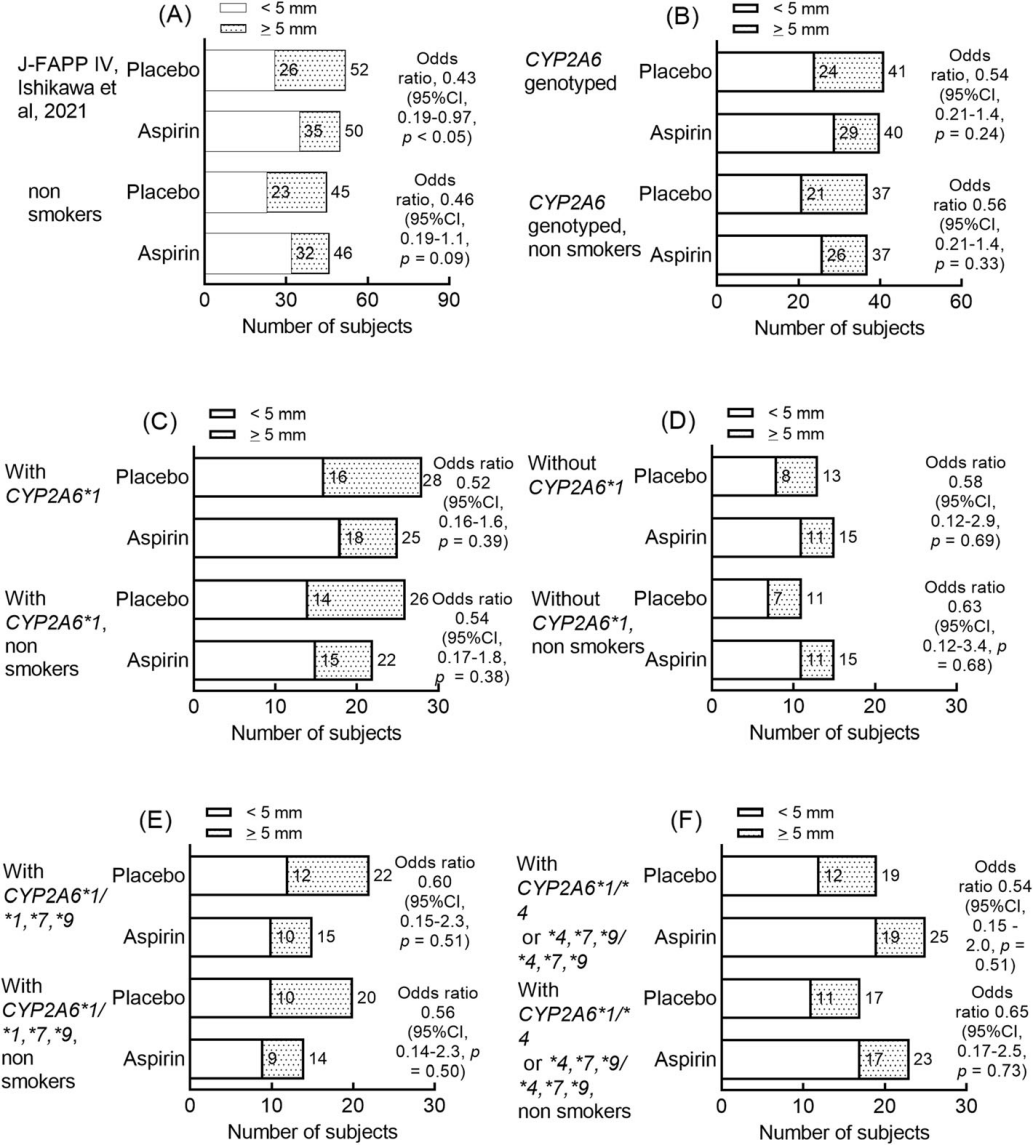
Effects of *CYP2A6* haplotypes and genotypes on aspirin chemoprevention for colorectal tumor recurrence in the total cohort and the nonsmoker subset of Japanese J-FAPP IV study participants. Data shown in Panel A were taken from Ishikawa et al. [15]. The preventive effects of aspirin were evaluated based on the numbers of polyps that had developed to a size of ≥5 mm (J-FAPP IV) observed after 8-months. Odds ratios are shown with respect to the reference (placebo) group

The chemopreventive effects of daily aspirin on the recurrence of colorectal tumors were also analyzed in Japanese cohorts in terms of polyps developing to a size of ≥5 mm (J-FAPP IV study) observed in the 8 months after endoscopic tumor excision. Significant chemopreventive effects of daily aspirin were observed in the whole cohort, with an odds ratio of 0.43 (95% CI, 0.19–0.97, Fig. 2a). Non-significant but favorable odds ratios were seen in the subset of J-FAPP IV participants who took part in the *CYP2A6* genotyping study (81 subjects, 8.6% smokers, odds ratio 0.54, 95% CI 0.2–1.4, Fig. 2b). Chemopreventive effects were found in subjects with the normal and with the impaired *CYP2A6* genotypes (Fig. 2c-f)*.* The chemopreventive effects of aspirin on colorectal tumor recurrence with apparent odds ratios of 0.52–0.65 were suggested in all the subsets of J-FAPP IV participants tested, under the reported negligible chemopreventive potential of mesalazine in the original findings [15].

## Discussion

Considerable evidence has been provided for potential chemoprevention of colorectal cancer by aspirin [10]. Collectively, when subjects with familial adenomatous polyposis were excluded, the presence of the wild-type allele of polymorphic *CYP2A6* apparently led to a reduction in the chemopreventive effects of daily aspirin on the sporadic development of colorectal tumors in nonsmokers (Fig. 1c, d). Moreover, although the mechanism is unknown, chemoprevention using daily aspirin to reduce the risk the colorectal tumors was found to be inversely dependent on the putative enzyme activity of the CYP2A6 phenotype (based on the presence/absence of CYP2A6*1 alleles) among a Japanese cohort without familial adenomatous polyposis (Fig. 1e, f), especially in nonsmoking men (Table 1). Wild-type *CYP2A6* was recently reported to be a risk index of arteriosclerosis as a lifestyle-related disease in the general Japanese population, although the mechanism is unknown [16].

The chemopreventive data from single-center subsets having daily aspirin from reported multicenter studies [9, 15] were reanalyzed with respect to variations in polymorphic CYP2A6. We were unable to analyze all the subjects by restricted ethical reasons. In the current study, because the number of subjects was relatively low and/or the endpoint was tumor recurrence, the entire population was evaluated with a possible limited confounding factor. However, it should be noted that this apparent limitation would yield a high accuracy in this study, because all colonoscopy diagnostics were consistently performed by single experienced physician with high adenoma detection rates.

## Conclusions

Consequently, the *CYP2A6* wild-type allele could be a potential biomarker candidate for reduced chemopreventive effects of daily aspirin in the Japanese population and could be applicable to future personalized treatments. Such tailored treatments would be particularly applicable in the Japanese population, which is known to have a wide range of CYP2A6 phenotypes, frequently including those with impaired activities caused by genetic variations and whole-gene deletions. Genotyping of the *CYP2A6* alleles could insight into future personalized chemoprevention with daily low-dose aspirin.

## Data Availability

All data generated or analyzed during this study are included in this published article and are also available from the corresponding author on reasonable request.

## Abbreviations

CYP2A6: Cytochrome P450 2A6
CI: Confidence interval
